# First record of *Leptoglossusoccidentalis* Heidemann, 1910 (Hemiptera, Coreidae) in the Canary Islands, a novel pine pest detected through citizen science in an oceanic archipelago

**DOI:** 10.3897/BDJ.11.e109851

**Published:** 2023-09-11

**Authors:** David Lugo, Daniel Suárez, Sonia Martín, Óscar Martín Afonso, Alicia Martín, Carlos Ruiz

**Affiliations:** 1 Departamento de Biología Animal, Edafología y Geología, Grupo de Sistemática, Biogeografía y Evolución de Artrópodos de Canarias, University of La Laguna, Tenerife, Canary Islands 38200, San Cristóbal de La Laguna, Spain Departamento de Biología Animal, Edafología y Geología, Grupo de Sistemática, Biogeografía y Evolución de Artrópodos de Canarias, University of La Laguna, Tenerife, Canary Islands 38200 San Cristóbal de La Laguna Spain; 2 Island Ecology and Evolution Research Group, Institute of Natural Products and Agrobiology (IPNA-CSIC), Astrofísico Francisco Sánchez 3, La Laguna, Tenerife, Canary Islands 38206, San Cristóbal de La Laguna, Spain Island Ecology and Evolution Research Group, Institute of Natural Products and Agrobiology (IPNA-CSIC), Astrofísico Francisco Sánchez 3, La Laguna, Tenerife, Canary Islands 38206 San Cristóbal de La Laguna Spain; 3 Servicio de Biodiversidad, Dirección General de Lucha contra el Cambio Climático y Medio Ambiente. Consejería de Transición Ecológica, Lucha contra el Cambio Climático y Planificación Territorial, Gobierno de Canarias, Las Palmas de Gran Canaria, Spain Servicio de Biodiversidad, Dirección General de Lucha contra el Cambio Climático y Medio Ambiente. Consejería de Transición Ecológica, Lucha contra el Cambio Climático y Planificación Territorial, Gobierno de Canarias Las Palmas de Gran Canaria Spain; 4 Gestión y Planeamiento Territorial y Medioambiental, Las Palmas de Gran Canaria, Spain Gestión y Planeamiento Territorial y Medioambiental Las Palmas de Gran Canaria Spain

**Keywords:** Coreidae, citizen science, Macaronesia, exotic species, DNA barcoding, new record

## Abstract

**Background:**

The 'western seed bug', known as *Leptoglossusoccidentalis*, is considered a global invasive species that has experienced a recent rapid expansion worldwide, becoming an important pest species for coniferous forests.

**New information:**

With the 'Canary Islands early-warning network for the detection and intervention of invasive exotic species' (RedEXOS), this species was detected for the first time in the Canarian archipelago in an urban area in the eastern part of the island of Gran Canaria. This early detection is crucial for understanding the potential damage in one of the islands with the highest surface area of natural endemic pine forest.

## Introduction

The genus *Leptoglossus* Guérin-Méneville, 1831 (Hemiptera, Coreidae, Anisoscelini) harbours up to 62 species with a Neotropical origin, most of them distributed in Central and South-America (Faúndez et al. 2017). The genus is characteried by a hind tibiae dilation that is lanceolate or conspicuously phylliform with one or more emarginations. In most cases, they are widely expanded, with a body surface scarcely pilose and the first antennal segment is equal to or longer than the head (Brailovsky and Barrera 2013). *Leptoglossusoccidentalis* Heidemann, 1910, also known as ‘American pine bug’ or ‘western conifer seed bug’, is native to the western part of North America, being present in Canada and the United States (Bánki et al. 2023). Currently, it is considered a global invasive species due to its spread across Europe, Asia, Africa and South America (Brailovsky 2014, Lesieur et al. 2014, Faúndez et al. 2017, Lesieur et al. 2018, van der Heyden 2019b, Carpintero et al. 2019,Carpintero et al. 2019). In Europe, it was first detected in northern Italy in 1999 (Taylor et al. 2001). Three years later, the species was present in the south of the country. In Spain, it was first reported in Barcelona in 2003 and quickly colonized the entire Iberian Peninsula, reaching the Atlantic coast in less than seven years in three independent events (Gallego et al. 2013, Farinha et al. 2023). In less than fifteen years, this species has colonized the entire European continent, with Norway being the northernmost point and Sicily the southernmost (Maltese et al. 2009, Mjøs et al. 2010). It is estimated that the species has expanded at a rate of 160 km/year, higher than the estimation of 90 km/year observed in the USA (Gall 1992). In South America, the first reports of the species were from Chile and Argentina in 2017 (Faúndez et al. 2017, Carpintero et al. 2019), followed by a recent expansion to Uruguay (2019), Brazil (2020) and Paraguay (2021) (Faúndez and Silvera 2019, van der Heyden and Faúndez 2020, Garcete-Barret et al. 2021). In Africa, the species was detected in Tunisia (2013), Morocco (2015) and South Africa (2020) (Jamâa et al. 2013, Gapon 2015, van der Heyden and Faúndez 2020, Giliomee and Rayner 2021). In Asia, it has been reported in Japan (Ishikawa and Kikuhara 2009), China and South Korea (Zhu 2010, Ahn et al. 2013). Additionally, the species was detected in the Middle East, with reports in Turkey (2011), Lebanon (2015) and Israel (2018) (Fent and Kment 2011, Nemer et al. 2019, van der Heyden 2019b).

The characteristic biology of this species (i.e. adults forming aggregations) has facilitated its human-mediated long-distance expansion, particularly through the transportation of large aggregations in commercial containers and other artificial structures. Adult specimens have been detected in containers with tree trunks and wood panels (Dusoulier et al. 2007, Faúndez et al. 2017). Specimens have also been observed near naval ports in areas such as Venice, Barcelona, Le Havre and Weymouth (Lesieur et al. 2014). This process also determines the pathways by which the species reaches islands, as observed in Sicily, Corsica, Great Britain, Mallorca, Malta, Crete or Menorca (Sciberras and Sciberras 2010, Pérez Valcárcel and Prieto Piloña 2010, Fent and Kment 2011, van der Heyden 2017, van der Heyden 2019a). Likewise, it possesses a remarkable capacity for flight, which is involved in dispersal and rapid expansion between territories, as observed in the Northern Sporades (Petrakis 2011, Langourov et al. 2022). Insular ecosystems harbour unique flora and fauna which are particularly vulnerable to the arrival of exotic species (Reaser et al. 2007). To date, the unique report of *L.occidentalis* for an oceanic island was made in 2013 on the sland of Madeira, where several specimens were collected from trees of *Pinuspinaster* Ait, 1789 and *Pinushalepensis* Mill, 1768 (Bella and Aguiar 2020). In the Canary Islands, another oceanic archipelago of the Macaronesian egion, 22 Coreidae species have been reported, with *Leptoglossusgonagra* (Fabricius, 1775) being the only introduced species from this family so far (Roca-Cusachs et al. 2020). Here, we report *L.occidentalis* for the first time for the Canary Islands, discuss potential threats for native biodiversity and the role of citizen science in the early detection of such invasion.

## Materials and methods

Specimens were identified from material collected in Gran Canaria and confirmed by DNA barcoding. Monitoring data have been obtained using the RedEXOS online platform (Gobierno de Canarias 2023), a freely available tool with smartphone application created by the Canary Island Government, as an early detection network specifically developed to detect invasive alien species. DNA was extracted from a specimen with the Mag-Bind Blood & Tissue DNA HDQ 96 kit (Omega Bio-Tek GA, USA) using a KingFisher Flex (Thermofisher) and DNA concentrations were measured using Tecan Infinite 200 Pro (Configuration: Infinite M Nano+). The 5’ region (658 bp) of the mtDNA COI gene was amplified using FoldF and FoldR primers (Yu et al. 2012). PCR reaction conditions were as follows: initial denaturation at 95°C for 4 min, followed by 42 cycles of 95°C for 45 s, 46°C for 45 s and 72°C for 90 s and a final extension of 72°C for 10 min. DNA extract (2 μl) was amplified with 23 μl of PCR mix (for a total volume of 25 μl), comprised of 14.4 μl of water, 2.5 μl of 10x NH_4_ buffer (Bioline), 1.5 μl of 50 mM MgCl_2_ (Bioline), 2 μl of 2.5 mM dNTPs (Bioline), 0.5 μl of BSA (20 mg/ml), 1 μl of each primer (10 μM) and 0.1 μl of Taq polymerase (BIOTAQ). PCR products were sequenced using the Sanger DNA sequencing service of Macrogen (www.macrogen.com). Sequences were then edited in Geneious 2021.1.1 (www.geneious.com) and compared to the ‘nucleotide’ database of GenBank (www.ncbi.nlm.nih.gov/genbank) using BLAST (Altschul et al. 1990). The identification was confirmed using the Barcode of Life Data Systems (BOLD) identification engine, which conducted a comprehensive search through the complete DNA barcode records in BOLD to find a suitable match. A dataset was assembled including our sequenced barcode and a set of available barcode sequences of *L.occidentalis*. A haplotype network was made using the median joining method (Bandelt et al. 1999) in Popart software (Leigh and Bryant 2015). The barcode sequence was deposited in GenBank (accession code OR268561).

## Taxon treatments

### Leptoglossus occidentalis

Heidemann, 1910

2D6A31D0-B935-57CD-89C7-018AC1982E25

#### Materials

- **Type status:** Other material. **Occurrence:** individualCount: 1; occurrenceID: 24BB4C7B-9818-5C17-A658-6676CD4B2EC4; **Taxon:** taxonID: https://www.gbif.org/es/species/5156102; scientificNameID: Leptoglossusoccidentalis; scientificName: Leptoglossusoccidentalis Heidemann, 1910; family: Coreidae; genus: Leptoglossus; specificEpithet: occidentalis; scientificNameAuthorship: Heidemann, 1910; **Location:** islandGroup: Canary Islands; island: Gran Canaria; country: Spain; stateProvince: Las Palmas; municipality: Las Palmas; locality: Tafira Alta; decimalLatitude: 28.0524757; decimalLongitude: -15.4659979; georeferenceProtocol: GPS; **Identification:** identifiedBy: Oscar Afonso; dateIdentified: 2023; **Event:** eventDate: 23/03/2023; eventRemarks: On floor
- **Type status:** Other material. **Occurrence:** individualCount: 1; occurrenceID: AA6B1220-B3FF-5F7C-AE72-57F41C88FB19; **Taxon:** taxonID: https://www.gbif.org/es/species/5156102; scientificNameID: Leptoglossusoccidentalis; scientificName: Leptoglossusoccidentalis Heidemann, 1910; family: Coreidae; genus: Leptoglossus; specificEpithet: occidentalis; scientificNameAuthorship: Heidemann, 1910; **Location:** islandGroup: Canary Islands; island: Gran Canaria; country: Spain; stateProvince: Las Palmas; municipality: Las Palmas; locality: Tafira Alta; decimalLatitude: 28.0524757; decimalLongitude: -15.4659979; georeferenceProtocol: GPS; **Identification:** identifiedBy: Oscar Afonso; dateIdentified: 2023; **Event:** eventDate: 03/16/2023; eventRemarks: On floor
- **Type status:** Other material. **Occurrence:** individualCount: 1; occurrenceID: 47CF4968-98C4-55B5-B3E4-24C92DF27E4A; **Taxon:** taxonID: https://www.gbif.org/es/species/5156102; scientificNameID: Leptoglossusoccidentalis; scientificName: Leptoglossusoccidentalis Heidemann, 1910; family: Coreidae; genus: Leptoglossus; specificEpithet: occidentalis; scientificNameAuthorship: Heidemann, 1910; **Location:** islandGroup: Canary Islands; island: Gran Canaria; country: Spain; stateProvince: Las Palmas; municipality: Las Palmas; locality: Tafira Alta; decimalLatitude: 28.0524757; decimalLongitude: -15.4659979; georeferenceProtocol: GPS; **Identification:** identifiedBy: Oscar Afonso; dateIdentified: 2023; **Event:** eventDate: 03/15/2023; eventRemarks: On floor
- **Type status:** Other material. **Occurrence:** individualCount: 1; occurrenceID: 3BD69B29-A18D-57B3-96E2-FD3781BBB995; **Taxon:** taxonID: https://www.gbif.org/es/species/5156102; scientificNameID: Leptoglossusoccidentalis; scientificName: Leptoglossusoccidentalis Heidemann, 1910; family: Coreidae; genus: Leptoglossus; specificEpithet: occidentalis; scientificNameAuthorship: Heidemann, 1910; **Location:** islandGroup: Canary Islands; island: Gran Canaria; country: Spain; stateProvince: Las Palmas; municipality: Las Palmas; locality: Tafira Alta; decimalLatitude: 28.0524757; decimalLongitude: -15.4659979; georeferenceProtocol: GPS; **Identification:** identifiedBy: Oscar Afonso; dateIdentified: 2023; **Event:** eventDate: 03/08/2023; eventRemarks: On floor
- **Type status:** Other material. **Occurrence:** individualCount: 1; occurrenceID: 0C358B4C-C079-5307-A5F9-A6EAAF9B3BED; **Taxon:** taxonID: https://www.gbif.org/es/species/5156102; scientificNameID: Leptoglossusoccidentalis; scientificName: Leptoglossusoccidentalis Heidemann, 1910; family: Coreidae; genus: Leptoglossus; specificEpithet: occidentalis; scientificNameAuthorship: Heidemann, 1910; **Location:** islandGroup: Canary Islands; island: Gran Canaria; country: Spain; stateProvince: Las Palmas; municipality: Las Palmas; locality: Tafira Alta; decimalLatitude: 28.0524757; decimalLongitude: -15.4659979; georeferenceProtocol: GPS; **Identification:** identifiedBy: Oscar Afonso; dateIdentified: 2023; **Event:** eventDate: 03/03/2023; eventRemarks: On floor
- **Type status:** Other material. **Occurrence:** individualCount: 1; occurrenceID: 6820D899-2233-5E6F-8905-6FBBF285F182; **Taxon:** taxonID: https://www.gbif.org/es/species/5156102; scientificNameID: Leptoglossusoccidentalis; scientificName: Leptoglossusoccidentalis Heidemann, 1910; family: Coreidae; genus: Leptoglossus; specificEpithet: occidentalis; scientificNameAuthorship: Heidemann, 1910; **Location:** islandGroup: Canary Islands; island: Gran Canaria; country: Spain; stateProvince: Las Palmas; municipality: Las Palmas; locality: Tafira Alta; decimalLatitude: 28.0524757; decimalLongitude: -15.4659979; georeferenceProtocol: GPS; **Identification:** identifiedBy: Oscar Afonso; dateIdentified: 2023; **Event:** eventDate: 03/01/2023; eventRemarks: On floor
- **Type status:** Other material. **Occurrence:** individualCount: 1; occurrenceID: 1F9BE725-E22F-5E08-9E31-4BDD8DBCF031; **Taxon:** taxonID: https://www.gbif.org/es/species/5156102; scientificNameID: Leptoglossusoccidentalis; scientificName: Leptoglossusoccidentalis Heidemann, 1910; family: Coreidae; genus: Leptoglossus; specificEpithet: occidentalis; scientificNameAuthorship: Heidemann, 1910; **Location:** islandGroup: Canary Islands; island: Gran Canaria; country: Spain; stateProvince: Las Palmas; municipality: Las Palmas; locality: Tafira Alta; decimalLatitude: 28.0524757; decimalLongitude: -15.4659979; georeferenceProtocol: GPS; **Identification:** identifiedBy: Oscar Afonso; dateIdentified: 2023; **Event:** eventDate: 02/14/2023; eventRemarks: On floor
- **Type status:** Other material. **Occurrence:** individualCount: 1; occurrenceID: 83B0A1F7-D767-5234-B066-D81688B7BFCD; **Taxon:** taxonID: https://www.gbif.org/es/species/5156102; scientificNameID: Leptoglossusoccidentalis; scientificName: Leptoglossusoccidentalis Heidemann, 1910; family: Coreidae; genus: Leptoglossus; specificEpithet: occidentalis; scientificNameAuthorship: Heidemann, 1910; **Location:** islandGroup: Canary Islands; island: Gran Canaria; country: Spain; stateProvince: Las Palmas; municipality: Las Palmas; locality: Tafira Alta; decimalLatitude: 28.0524757; decimalLongitude: -15.4659979; georeferenceProtocol: GPS; **Identification:** identifiedBy: Oscar Afonso; dateIdentified: 2023; **Event:** eventDate: 02/14/2023; eventRemarks: On floor
- **Type status:** Other material. **Occurrence:** individualCount: 1; occurrenceID: 88CDAA00-8ABB-5E75-AEFF-C7ECE6AFB6B4; **Taxon:** taxonID: https://www.gbif.org/es/species/5156102; scientificNameID: Leptoglossusoccidentalis; scientificName: Leptoglossusoccidentalis Heidemann, 1910; family: Coreidae; genus: Leptoglossus; specificEpithet: occidentalis; scientificNameAuthorship: Heidemann, 1910; **Location:** islandGroup: Canary Islands; island: Gran Canaria; country: Spain; stateProvince: Las Palmas; municipality: Las Palmas; locality: Tafira Alta; decimalLatitude: 28.0524757; decimalLongitude: -15.4659979; georeferenceProtocol: GPS; **Identification:** identifiedBy: Oscar Afonso; dateIdentified: 2022; **Event:** eventDate: 12/20/2022; eventRemarks: On floor
- **Type status:** Other material. **Occurrence:** individualCount: 1; occurrenceID: A7194FAB-F818-5CD2-A28F-2A71D8C3A089; **Taxon:** taxonID: https://www.gbif.org/es/species/5156102; scientificNameID: Leptoglossusoccidentalis; scientificName: Leptoglossusoccidentalis Heidemann, 1910; family: Coreidae; genus: Leptoglossus; specificEpithet: occidentalis; scientificNameAuthorship: Heidemann, 1910; **Location:** islandGroup: Canary Islands; island: Gran Canaria; country: Spain; stateProvince: Las Palmas; municipality: Las Palmas; locality: Tafira Alta; decimalLatitude: 28.0524757; decimalLongitude: -15.4659979; georeferenceProtocol: GPS; **Identification:** identifiedBy: Oscar Afonso; dateIdentified: 2022; **Event:** eventDate: 11/18/2022; eventRemarks: On floor
- **Type status:** Other material. **Occurrence:** individualCount: 1; occurrenceID: B60A8D20-9495-50A0-B910-251E2A80C92C; **Taxon:** taxonID: https://www.gbif.org/es/species/5156102; scientificNameID: Leptoglossusoccidentalis; scientificName: Leptoglossusoccidentalis Heidemann, 1910; family: Coreidae; genus: Leptoglossus; specificEpithet: occidentalis; scientificNameAuthorship: Heidemann, 1910; **Location:** islandGroup: Canary Islands; island: Gran Canaria; country: Spain; stateProvince: Las Palmas; municipality: Las Palmas; locality: Tafira Alta; decimalLatitude: 28.0524757; decimalLongitude: -15.4659979; georeferenceProtocol: GPS; **Identification:** identifiedBy: Oscar Afonso; dateIdentified: 2022; **Event:** eventDate: 11/02/2022; eventRemarks: On floor
- **Type status:** Other material. **Occurrence:** individualCount: 3; occurrenceID: 88270BC1-3CB6-5716-89EB-F77331E05D74; **Taxon:** taxonID: https://www.gbif.org/es/species/5156102; scientificNameID: Leptoglossusoccidentalis; scientificName: Leptoglossusoccidentalis Heidemann, 1910; family: Coreidae; genus: Leptoglossus; specificEpithet: occidentalis; scientificNameAuthorship: Heidemann, 1910; **Location:** islandGroup: Canary Islands; island: Gran Canaria; country: Spain; stateProvince: Las Palmas; municipality: Las Palmas; locality: Tafira Alta; decimalLatitude: 28.0524757; decimalLongitude: -15.4659979; georeferenceProtocol: GPS; **Identification:** identifiedBy: Oscar Afonso; dateIdentified: 2022; **Event:** eventDate: 10/28/2022; eventRemarks: On floor
- **Type status:** Other material. **Occurrence:** individualCount: 1; occurrenceID: FE9FB03B-1995-5459-9D84-71DBC725F133; **Taxon:** taxonID: https://www.gbif.org/es/species/5156102; scientificNameID: Leptoglossusoccidentalis; scientificName: Leptoglossusoccidentalis Heidemann, 1910; family: Coreidae; genus: Leptoglossus; specificEpithet: occidentalis; scientificNameAuthorship: Heidemann, 1910; **Location:** islandGroup: Canary Islands; island: Gran Canaria; country: Spain; stateProvince: Las Palmas; municipality: Las Palmas; locality: Tafira Alta; decimalLatitude: 28.0524757; decimalLongitude: -15.4659979; georeferenceProtocol: GPS; **Identification:** identifiedBy: Oscar Afonso; dateIdentified: 2022; **Event:** eventDate: 10/25/2022; eventRemarks: On floor
- **Type status:** Other material. **Occurrence:** individualCount: 4; occurrenceID: 5A0FA7FA-A33C-53CE-8787-5BE30839F104; **Taxon:** taxonID: https://www.gbif.org/es/species/5156102; scientificNameID: Leptoglossusoccidentalis; scientificName: Leptoglossusoccidentalis Heidemann, 1910; family: Coreidae; genus: Leptoglossus; specificEpithet: occidentalis; scientificNameAuthorship: Heidemann, 1910; **Location:** islandGroup: Canary Islands; island: Gran Canaria; country: Spain; stateProvince: Las Palmas; municipality: Las Palmas; locality: Tafira Alta; decimalLatitude: 28.0524757; decimalLongitude: -15.4659979; georeferenceProtocol: GPS; **Identification:** identifiedBy: Oscar Afonso; dateIdentified: 2022; **Event:** eventDate: 11/29/2022; eventRemarks: On floor
- **Type status:** Other material. **Occurrence:** individualCount: 1; occurrenceID: 341CFD2E-1E2F-5083-9878-014687D7EEFC; **Taxon:** taxonID: https://www.gbif.org/es/species/5156102; scientificNameID: Leptoglossusoccidentalis; scientificName: Leptoglossusoccidentalis Heidemann, 1910; family: Coreidae; genus: Leptoglossus; specificEpithet: occidentalis; scientificNameAuthorship: Heidemann, 1910; **Location:** islandGroup: Canary Islands; island: Gran Canaria; country: Spain; stateProvince: Las Palmas; municipality: Las Palmas; locality: Tafira Alta; decimalLatitude: 28.0524757; decimalLongitude: -15.4659979; georeferenceProtocol: GPS; **Identification:** identifiedBy: Heriberto Lopez; dateIdentified: 2022; **Event:** eventDate: 10/05/2022; eventRemarks: On floor; **Record Level:** institutionID: IPNA-CSIC; collectionID: BC1481

#### Diagnosis

**Figure 1.**
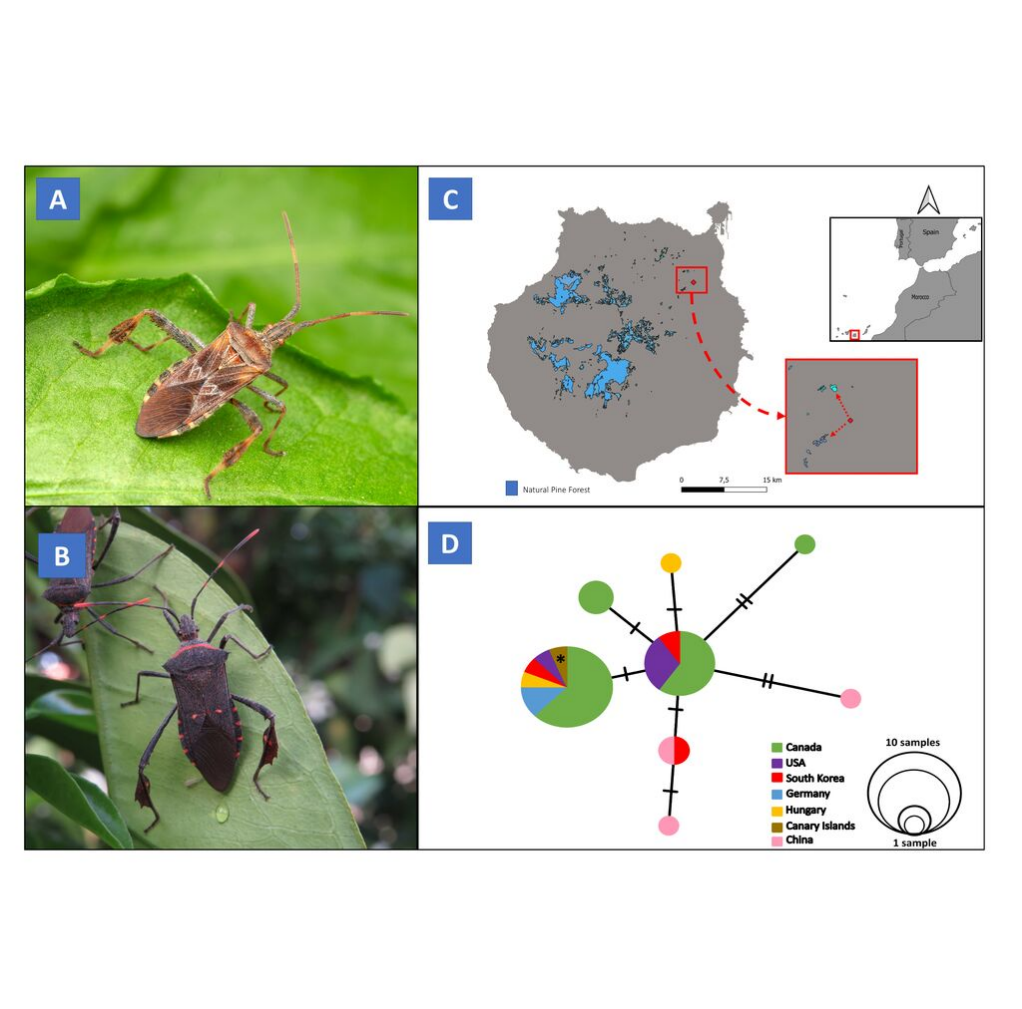
Habitus, distribution and genetic data of *Leptoglossusoccidentalis*. **A** Dorsal photo of *L.occidentalis*, with reduced dilatations in the hind tibias (photo: Manuel Arechavaleta). **B** Dorsal photo of *L.gonagra* with marked orange dots and teeth in the hind tibia´s dilatations (photo: Daniel Suárez). **C** Map of the sland of Gran Canaria showing the presence of *Leptoglossusoccidentalis* (red dot) and the distribution of Pine forest of Gran Canaria (blue). Nearby pine cores (2 km) are marked with red arrows in the red inset. The location of Gran Canaria within the Canary Islands is marked with a red square in the black inset. **D** Haplotype network for *L.occidentalis*, based on sequence variation of the COI barcode region. An asterisk indicates the haplotype from Gran Canaria.

Adult specimens measure between 1.5 and 2.5 cm (total length) and have a brown or black body colouration. It can be distinguished from *L.gonagra* by its broadly rounded humeral angles of the pronotum, a brown pronotal disc with several round black spots, underside of body without contrasting large yellow spots and smaller and narrower foliar dilations of the tibiae (Fig. 1A). In contrast, *L.gonagra* has humeral angles of the pronotum that end in a spine, an orange line crossing the anterior part of the pronotal disc, underside of body covered with large contrasting yellow spots and larger and wider leaf-shaped dilations of the posterior tibiae with teeth on the external part (Fig. 1B).

#### Distribution

North America, Canada, United States, South America, Europe, Asia and Africa (Brailovsky 2014, Lesieur et al. 2014, Faúndez et al. 2017, Carpintero et al. 2019, van der Heyden and Faúndez 2020, Bánki et al. 2023).

##### Habitat

The current distribution of the species can be found at Fig. 1C. The first individual was detected in Tafira Alta, Las Palmas de Gran Canaria (28.0524757, -15.465979), in early October 2022. Since then, the locality has been monitored by technicians during five months and fourteen additional individuals have been found (see 'Materials and Methods').

##### Genetic data

A 658-bp fragment was succesfully amplified (GenBank accession code: OR268561). BOLD identification resulted at species level identification with a 100% correlation, with the specimen from Gran Canaria sharing haplotype with continental specimens from Canada, Germany, Hungary, USA and South Korea (Fig. 1D).

## Discussion

In their native area, the life cycle of *L.occidentalis* is univoltine, forming aggregations to hibernate in autumn (Pérez Valcárcel and Prieto Piloña 2010). These can be found under the bark of logs, in cracks or in human-made structures. They can also become a nuisance pest in houses by entering through small cracks and crevices in buildings (Gall 1992). However, outside their native range, the species has become bivoltine and polyvoltine, with a peak of activity in October and December, when aggregations search for hibernation shelters (Pérez Valcárcel and Prieto Piloña 2010).

This species lays eggs on the needles of conifers. It primarily feeds on the reproductive structures of different conifer species, such as pines (*Pinus* spp.), spruces (*Picea* spp.) and firs (*Abies* spp.), causing a reduction in fertility. This can occur through the induction of abortion in the early stages of development (Connelly and Schowalter 1991) or the damage mature pine cones, resulting in a lack of germination (Bates et al. 2001, Bates et al. 2002, Lesieur et al. 2014). Adults and nymphs may occasionally puncture plant tissues to access the xylem and hydrate themselves, leading to damage to needles, bark and branches. Due to their ability to feed on different species of pine trees and the damage they cause to commercially valuable species, there is concern about their impact on the endemic ‘Canary pine’ (*Pinuscanariensis* C. Sm. ex DC. in Buch). The recent arrival and establishment of this species could lead to a significant reduction in fertility and the seed bank of the ‘Canary pine’. Currently, *L.occidentalis* appears to be located outside the core areas of the natural pine forest, but close to fragment areas that could connect with the core areas (Fig. 1C). Due to their high dispersal abilities, there is a great possibility for this species to spread throughout the Island. Additionally, its aggregation behaviour and its human-mediated long-distance expansion could enhance its spread across the Archipelago. Continuing with monitoring is thus crucial, as the deterioration of the Island's natural pine forest ecosystems can have cascading effects. *Leptoglossusoccidentalis* may directly impact on Canary pine seeds, depriving food resources for other native species, such as the ‘Gran Canaria Blue Chaffinch’ (*Fringillapolatzeki* Hartert, 1905). This bird species is listed as ‘in danger of extinction’ in the Catalogue of Threatened Species of the Canary Islands and on the IUCN Red List. It is also considered a priority species in Annex I of the Birds Directive 79/409/EEC.

The role of citizen science as a tool for early detection is crucial in order to make appropriate and successful control efforts and reduce considerable time in eradication (Larson et al. 2020). Institutional proposals, such as the Canary Island Government with the RedEXOS online platform, are key to preventing uncontrolled expansions and subsequent unnoticed colonisations (e.g. Rodríguez et al. (2021), Scholz et al. (2021)). Any suspicious or unusual location of unknown species in the territory should alert institutions and experts in biological invasions (Thomas et al. 2017). The role of both citizen science through the use of the RedEXOS app, as well as a pertinent institution to monitor the current distribution and to sample potential habitat areas on the Island, is crucial in order to assess the current distribution of the species, which can expand hundreds of kilometres each year without difficulty.

## Supplementary Material

XML Treatment for Leptoglossus occidentalis
